# Circulating biomarkers associated with placental dysfunction and their utility for predicting fetal growth restriction

**DOI:** 10.1042/CS20220300

**Published:** 2023-04-19

**Authors:** Jesrine Hong, Sailesh Kumar

**Affiliations:** 1 Mater Research Institute, University of Queensland, Level 3, Aubigny Place, Raymond Terrace, South Brisbane, Queensland 4101, Australia; 2 Department of Obstetrics and Gynecology, Faculty of Medicine, Universiti Malaya, Kuala Lumpur 50603, Malaysia; 3 School of Medicine, The University of Queensland, Herston, Queensland 4006, Australia

**Keywords:** Circulating biomarkers, Fetal growth restriction, Placental dysfunction, Small for gestational age

## Abstract

Fetal growth restriction (FGR) leading to low birth weight (LBW) is a major cause of neonatal morbidity and mortality worldwide. Normal placental development involves a series of highly regulated processes involving a multitude of hormones, transcription factors, and cell lineages. Failure to achieve this leads to placental dysfunction and related placental diseases such as pre-clampsia and FGR. Early recognition of at-risk pregnancies is important because careful maternal and fetal surveillance can potentially prevent adverse maternal and perinatal outcomes by judicious pregnancy surveillance and careful timing of birth. Given the association between a variety of circulating maternal biomarkers, adverse pregnancy, and perinatal outcomes, screening tests based on these biomarkers, incorporating maternal characteristics, fetal biophysical or circulatory variables have been developed. However, their clinical utility has yet to be proven. Of the current biomarkers, placental growth factor and soluble fms-like tyrosine kinase 1 appear to have the most promise for placental dysfunction and predictive utility for FGR.

## Introduction

A small for gestational age (SGA) infant is variably defined as one with an estimated fetal weight (EFW) or birthweight (BW) less than the 10^th^ centile for gestation [1–3]. Globally, almost 21 million infants are born SGA each year, the majority in low-income and middle-income countries [4]. These infants are at higher risk of morbidity and mortality particularly if they are born preterm [5] and are also more likely to develop chronic health complications through childhood and in adulthood [6].

Although SGA and fetal growth restriction (FGR) are often used interchangeably, FGR is defined as an infant that has not achieved its genetic growth potential [7]; however, as this is inherently unknown, it is impossible to determine if any infant has indeed achieved that potential prenatally. Importantly, not all infants with FGR will be SGA and not all SGA infants will have FGR. Regardless of this caveat, many SGA/FGR liveborn infants will have low birth weight (LBW) defined as a BW <2500 g irrespective of gestational age. Worldwide, LBW is an important public health indicator, especially in settings where accurate gestational age assessment is not possible and prenatal assessment of fetal size or growth is not available [5,8].

Normal placental development [9] involves a combination of highly regulated processes requiring a plethora of angiogenic growth factors, hormones, transcription factors, cytokines and cell adhesion molecules [10]. Failure to establish a high capacitance, low pressure maternal fetal vascular interface [9] leads to placental dysfunction and is causally related to several obstetric syndromes including pre-eclampsia and FGR [11]. Although there is considerable overlap between the pathogenesis of pre-eclampsia and FGR, the relationship between the extent of failure of spiral artery conversion, gestation at onset, type of disease, maternal and infant phenotype as well as clinical outcomes remains poorly understood [12–14].

The challenge obstetricians face is identifying the truly growth restricted fetus regardless of size, as these are the infants that are most at risk of adverse outcomes. Because placental dysfunction leading to inadequate nutrient and oxygen transfer [15] accounts for the majority of SGA/FGR infants [16,17], many investigators have focused attention on circulating biomarkers indicative of aberrant placental function [18–20]. As there is currently no treatment for placental dysfunction, early recognition of at-risk pregnancies is important because careful maternal and fetal surveillance can be instituted and adverse outcomes potentially prevented by judicious timing of birth [21–23]. The aim of this narrative review is to provide an overview of available evidence regarding circulating biomarkers associated with placental dysfunction and to discuss their clinical utility for the prediction of FGR. A comprehensive review of PubMed, Cochrane Library, and CINAHL was performed to identify appropriate publications between 1995 and October 2022 relevant to this review.

**Figure 1:**
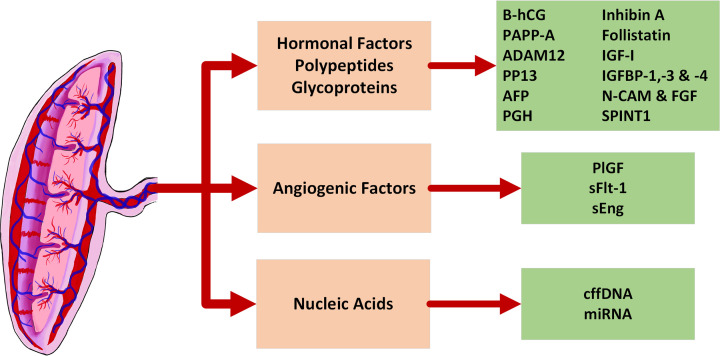
Circulating maternal biomarkers derived from the placenta that are associated in placental dysfunction A summary of various hormonal factors, polypeptides, glycoproteins, angiogenic factors, and nucleic acids, which are associated in placental dysfunction and pathophysiology of FGR.

Circulating biomarkers are broadly classified into: (1) hormonal factors, polypeptides, and glycoproteins; (2) angiogenic factors; and (3) cell-free nucleic acids [12,24] (Figure 1). Some of these biomarkers are potentially expressed as a consequence of epigenetic changes during placental development [25–28].

## Hormonal factors, polypeptides, and glycoproteins

Table 1 lists various placental hormones, polypeptides, and glycoproteins associated with placental dysfunction and their potential roles for screening and diagnosis of FGR. These include beta-human chorionic gonadotrophin (β-hCG), pregnancy-associated plasma protein-A (PAPP-A), A Disintegrin and Metalloprotease 12 (ADAM12), placental protein 13 (PP13), alpha-fetoprotein (AFP), inhibin A, activin A, follistatin, placental growth hormone (PGH), neural cell adhesion molecule (N-CAM), fibroblast growth factor (FGF), Insulin-like growth factor-I (IGF-I), Insulin-like growth factor binding proteins-1, -3, -4 (IGFBP-1, IGFBP-3, and IGFBP-4), and serine protease inhibitor Kunitz type 1 (SPINT1).

**Table 1:** Circulating maternal biomarkers associated with FGR

Biomarkers	Functions	Maternal levels in FGR pregnancies	Key references
**Hormonal factors, polypeptides, and glycoproteins**			
Activin A	Regulation of endometrial receptivity, implantation of embryo, and trophoblast development	Unchanged	[51]
		Raised	[50]
ADAM12^a^	Promotion of cell migration and trophoblast invasion	Reduced	[29,31,32,35,37]
AFP^b^	Function in human placenta is unclear	Raised in first and second trimester	[34,46,47]
		Reduced in third trimester	[48]
β-HCG^c^	Promotion of progesterone production by corpus luteal cells and maintenance of endometrial lining. Promotion of angiogenesis, immunosuppression, and growth of fetus organs	Reduced in first trimester	[30,32,37]
		Unchanged in second trimester	[34]
Follistatin	Inhibits the biological activity of Activin A. Inhibits follicular development in ovary by antagonizing follicle-stimulating hormone	Reduced	[50,51]
Inhibin A	Regulation of implantation and differentiation of developing embryo	Unchanged	[51–53]
		Raised	[50]
IGF-I^d^	Promotion of transplacental nutrient transfer to the fetus	Reduced	[58,61]
IGFBP-1^e^	Regulation of implantation and endometrial growth	Reduced	[58,59]
IGFBP-3^e^	Modulation of IGF-I effect in transplacental nutrient transfer	Unchanged	[58]
IGFBP-4^e^	Regulation of IGF bioavailability	Raised	[60]
N-CAM^f^	Cell signaling, adhesion, proliferation, and differentiation in fetal development. Maintenance of tissue integrity and regeneration of neural and non-neural tissues during early development of fetus	Increased	[61]
PGH^g^	Regulation of placental and fetal growth. Stimulation of IGF-I secretion	Unchanged	[57]
PP13^h^	Regulation of implantation and placental vascular development	Unchanged	[33,37,40–42,150]
		Reduced	[43]
PAPP-A^i^	Interaction with IGF and regulation of trophoblast and fetal growth	Reduced	[19,29,30,32,34,37]
SPINT1^j^	Mediates secretion of trophoblast degradative enzymes that regulate invasion and remodeling of endometrial spiral arteries	Reduced	[68–70]
**Angiogenic factors**			
PlGF^k^	Angiogenic factor expressed in villous syncytiotrophoblast to promote development and maturation of placental vascular system	Reduced	[37,48,76,79,80,86,91,92,94–98,107,151–157]
		Increased	[158,159]
		Unchanged	[160,161]
sFlt-1^l^	Antiangiogenic protein that antagonizes the actions of vascular endothelial growth factor and placental growth factor	Increased	[48,80,86,91,92,94–98,152–154,156–158,162–166]
		Reduced	[151,166,159]
		Unchanged	[107,155,160,167]
VEGF-A^m^	Promotes placental vasculogenesis and angiogenesis throughout pregnancy by promoting formation of angioblasts and mesenchymal villi	Increased	[158]
sEng^n^	Inhibits transforming growth factor beta (TGF-β)-mediated cell signaling and endothelial function	Increased	[111,112,166,168]
		Unchanged	[113,158]
FGF^o^	Regulates placental growth, differentiation, and angiogenesis	Increased	[61]
		Reduced	[63]

a A Disintegrin and Metalloprotease 12.

b Alpha-fetoprotein.

c Beta-human chorionic gonadotrophin.

d Insulin-like growth factor-I.

e Insulin-like growth factor binding proteins-1, -3, and -4.

f Neural cell adhesion molecule.

g Placental growth hormone.

h Placental protein 13.

I Pregnancy-associated plasma protein-A.

j Serine protease inhibitor Kunitz type 1.

k Placental growth factor.

l Soluble fms-like tyrosine kinase 1.

m Vascular endothelial growth factor-A.

n Soluble endoglin.

o Fibroblast growth factor.

### βhCG, PAPP-A, and ADAM12

Lower concentrations of circulating maternal βhCG, PAPP-A, and ADAM12 measured at 11–14 weeks of gestation have been reported in women with SGA/FGR infants [29–33]. Pihl et al. observed that first trimester maternal serum concentrations of βhCG, PAPP-A, and ADAM12 in women with SGA infants (defined as BW <5^th^ centile) were significantly lower compared with matched controls (βhCG: 0.74 vs. 1.04 multiples of median (MoM), PAPP-A: 0.64 vs. 1.02 MoM and ADAM12: 0.74 vs. 0.97 MoM). Combining βhCG and PAPP-A yielded a detection rate of 26% with a false-positive rate (FPR) of 5% for an SGA infant. However, the addition of ADAM12 only very modestly improved the detection rate by a further 2% [32]. In another study, Poon et al. found that first trimester βhCG and PAPP-A concentrations in combination with maternal characteristics and fetal nuchal translucency measurement predicted birth of an SGA infant [30]. Maternal βhCG and PAPP-A MoM were significantly lower in SGA pregnancies, and combining maternal factors, nuchal translucency thickness, PAPP-A, and free βhCG concentrations resulted in the highest area under receiver-operating curve (AUROC) of 0.747 (95% CI: 0.735–0.760) and a detection rate of 37% for a FPR of 10% [30]. In contrast, however, a screening test later in pregnancy using a similar combination of biomarkers, maternal factors, and fetal biometry at 19–24 weeks of gestation performed poorly for the prediction of an SGA infant [34].

Other studies have shown that although there is good correlation between first trimester maternal serum ADAM12 concentrations with BW centile, it performs poorly as a standalone screening test [29]. A more recent study evaluated maternal plasma ADAM12 late in the third trimester (36 weeks) and despite finding significantly lower median concentrations in women with SGA infants compared with controls [14115 pg/ml (Interquartile range (IQR): 11510–16592 pg/ml) vs. 16582 pg/ml (IQR: 13658–20322 pg/ml)], it was not suitable as a screening test [35].

A systematic review and meta-analysis (32 studies; 175240 pregnancies) assessing the predictive utility of first trimester maternal serum PAPP-A concentrations for birth of an SGA infant, revealed poor predictive value with low positive (PLR) and negative (NLR) likelihood ratios for BW < 10^th^ centile: PLR 1.96 (95% CI: 1.58–2.43), NLR 0.93 (95% CI: 0.89–0.98); BW < 5^th^ centile: PLR 2.65 (95% CI: 2.35–2.99), NLR 0.85 (95% CI: 0.74–0.98) [36], suggesting that PAPP-A was not suitable as a standalone biomarker to predict SGA infants [19,36]. In another case–control study at 11–13 weeks of gestation, a combination of uterine artery pulsatility index (UtA-PI), maternal mean arterial pressure (MAP), and serum concentrations of PAPP-A, βhCG, PlGF, PP13, ADAM12 and fetal nuchal translucency thickness, yielded a detection rate of 73% and 46% for birth of a preterm and term SGA infant, respectively [37].

### PP13 and AFP

PP13 is a glycan-binding protein mainly expressed in syncytiotrophoblast and secreted into the maternal circulation via exosomes or microvesicles [38]. Although an earlier observational study showed an association between low first trimester PP13 concentrations in maternal serum and FGR [39], subsequent studies [33,40] found limited predictive utility with no significant differences in median PP13 MoM levels in FGR-affected pregnancies even when combined with other first trimester screening markers such as PAPP-A [33] and ADAM12 [40]. Another study also showed that median PP13 concentrations in the first trimester of women with SGA infants (BW < 3^rd^, < 5^th^, and < 10^th^ centiles) were not significantly lower than the control arms (0.978, 1.058, 1.051, and 1.083 MoM (controls) for each BW centiles, respectively) [41]. These findings were further corroborated in another study [42], which also failed to demonstrate the utility of PP13 for prediction of FGR [41]. In another study, median first trimester PP13 concentrations were significantly lower in FGR pregnancies compared with controls (86.6 vs. 132.5 pg/ml) but the overall sensitivity for the prediction of FGR was low at 33% at a specificity rate of 90% [43]. Two systematic reviews of first trimester serum PP13 in combination with maternal characteristics for the prediction of SGA infants reported low sensitivity of 32% (95% CI: 18–48%) [44] and 36% (95% CI: 33–41%) [45], respectively. The overall evidence thus far suggests that PP13 has limited clinical utility for predicting FGR.

In a study of 9715 singleton pregnancies (including 481 SGA infants with BW < 5^th^ percentile), higher mean log_10_ MoM value of maternal serum AFP at 19–24 weeks of gestation was seen in the SGA cohort. When AFP levels were combined with maternal factors, fetal biometry, and maternal PlGF concentrations, detection rates of 100%, 76%, and 38% were achieved for SGA infants delivered at <32, 32–36, and ≥37 weeks gestation, respectively [34]. The addition of UtA-PI measurement further improved detection rates of SGA infants to 78% at 32–36 weeks and 42% at >37 weeks, respectively [46]. Another retrospective study [47] showed that while elevated serum AFP concentrations (≥2.5 MoM) in the first trimester was associated with birth of an SGA infant, its predictive utility for SGA and FGR was low with an AUROC of <0.6 [47]. Similarly, other studies have also demonstrated that although third trimester AFP concentrations are significantly lower in women with SGA infants, the overall detection rate using this biomarker is low at 26%, and even when it is combined with maternal PlGF concentrations detection rates only modestly increase to 32% [48]. A recent meta-analysis (39 cohort studies; 93968 women) reported that the relative risk (RR) for birth of an SGA infant in women with elevated AFP concentrations was increased (RR: 2.02, 95% CI: 1.75–2.33) and this risk was higher when ultrasound evidence of SGA was present (RR: 5.28, 95% CI: 3.46–8.06) [49].

### Inhibin A, activin A, and follistatin

Maternal serum concentrations of activin A, inhibin A, and the activin:follistatin ratio in the third trimester have been reported to be significantly increased in FGR pregnancies compared with controls [50]. However, in another study, there was no difference in activin A or inhibin A concentrations in normotensive women with SGA infants compared with controls [51]. A study by Miranda et al. [52] showed that although mean inhibin A concentrations were significantly higher in women with SGA infants, a multivariable integrative model of maternal characteristics, fetoplacental ultrasound, and maternal biochemical markers only modestly improved the detection of SGA/FGR cases at 32–36 weeks’ gestation when compared with screening based on EFW centiles alone. Other studies [53] have also shown that the clinical utility of activin A, inhibin A, and follistatin as predictors for SGA/FGR is poor.

### PGH, IGF-I, IGFBP, N-CAM, and FGF

PGH is mainly expressed by syncytiotrophoblast and stimulates gluconeogenesis and anabolic pathways to support the growing fetoplacental unit [54]. Earlier observational studies reported an association between low maternal serum PGH concentrations in the second and third trimesters and birth of an SGA infant [55,56]. However, in a later study [57], first trimester median maternal serum PGH concentrations in SGA pregnancies were not different to controls (0.95 MoM, 95% CI: 0.60–1.30 vs. 1.00 MoM, 95% CI: 0.70–1.30) and there was no association with BW centile.

Another study reported that although median maternal serum concentrations of IGF-I (61.8 ng/ml, IQR: 43.4–93.4 vs. 94.9 ng/ml, IQR: 56.7–131.2), IGFBP-1 (58.2 ng/ml, IQR: 39.8–84.9 vs. 81.4 ng/ml, IQR: 57.3–105.5), and IGFBP-3 (54.5 ng/ml, IQR: 45.6–61.5 vs. 55.4 ng/ml, IQR: 47.4–64.9) were significantly lower in women with SGA infants compared with controls [58], after multiple regression analyses and adjustment for maternal characteristics, these biomarkers were ultimately not useful for the prediction of SGA. Similarly, in another study, although a significant negative correlation between log IGFBP-1 and BW standard deviation score was noted, after adjusting for maternal body mass index, the relationship became nonsignificant [59]. IGFBP-4 is highly expressed by extravillous trophoblasts at the maternal–fetal interface [60] and circulating maternal IGFBP-4 concentrations in early pregnancy have been reported to be significantly higher in women with FGR infants (defined as BW < 5^th^ centile) compared with controls [Odds ratio (OR) 22.3, (95% CI: 2.7–181.5)] with 93% positive predictive value (PPV) [60]. Current evidence, however, does not support the use of PGH, IGF-I, or IGFBP as reliable markers for the prenatal prediction of FGR [57–60].

A small observational study [61] reported an association between increased placental expression of N-CAM and FGF in cytotrophoblasts of pregnancies complicated by SGA (N-CAM immunoreactive cells median [range]: 26.0 [8–110] vs. 15.0 [8–29] (control group) and FGF: 45.0 [18–36] vs. 14.5 [5–26]). Another study showed that FGF-21 concentrations were significantly increased in amniotic fluid of SGA/FGR fetuses [62]. However, Hill et al. found that although maternal serum immunoreactive FGF-2 concentrations were lower in SGA pregnancies as compared with controls, the differences were not statistically significant [63]. The available evidence so far for the use of N-CAM and FGF for prediction of SGA/FGR is limited, and thus they should not be used in clinical practice until more data are available.

A systematic review and meta-analysis (103 studies; 432621 women) evaluating first trimester biomarkers (PAPP-A, βhCG, PlGF, and PP13) for the prediction of SGA reported low overall predictive accuracy [45]. Another review of AFP, βhCG, unconjugated estriol, PAPP-A, and inhibin A measured before 25 weeks gestation also reported poor predictive utility for SGA for all analytes [64]. However, high AFP and βhCG concentrations (>2 or >2.5 MoM) combined, appears to have better predictive utility for SGA infants (PLR: 6.18; 95% CI: 1.84–20.85) compared with unconjugated estriol, PAPP-A, and inhibin A [64,65]. Overall, however, the predictive value of AFP, βhCG, unconjugated estriol, PAPP-A, and inhibin A as biomarkers for SGA/FGR is low, either separately or in combination or incorporating maternal characteristics or ultrasound fetal biophysical variables [36,44,45,64–66].

### SPINT1

SPINT1 is a circulating protein highly expressed by villous cytotrophoblasts. It is involved in the conversion of maternal spiral arteries into low pressure, high capacitance vessels by modulating trophoblast secretion of proteolytic enzymes (serine proteinases, metalloproteinases, and collagenases) that regulate transformation of spiral arteries in normal placentation [67]. *In vitro* and animal studies suggest that SPINT1 is modulated by hypoxia and decreased in FGR placentae. In a study of 2003 women [68] at 36 weeks’ gestation, a strong association between low plasma SPINT1 concentrations and SGA (defined as <10^th^ centile) was seen. Using a SPINT1 cutoff threshold of <0.63 MoM, the risk of delivering an SGA infant with BW <3^rd^, <5^th^, and <10^th^ centile was 14.1%, 19.7%, and 28.2%, respectively [68]. A recent cohort study [69] found maternal plasma SPINT1 concentrations were significantly lower at 20 weeks gestation in women who subsequently delivered an SGA infant; however, the AUROC was modest at 0.62 for BW <3^rd^ centile and 0.56 for BW <19^th^ centile, respectively. Murphy et al. [70] reported reduced plasma SPINT1 concentration in women with pre-eclampsia who subsequently delivered an SGA infant (median [IQR]: 18857 pg/ml [10782–29890] in SGA vs. 40168 pg/ml [22172342–75] in controls). Another study by Murphy et al. [67] found an association between elevated plasma serine protease inhibitor Kunitz type 2 (SPINT2), which is functionally related to SPINT1 in pregnancies complicated by pre-eclampsia and/or SGA. However, the evidence for SPINT1 as a suitable biomarker to predict SGA/FGR is limited, and more evidence is required to validate its clinical utility.

## Angiogenic factors

Table 1 presents angiogenic biomarkers associated with SGA or FGR. Both vascular endothelial growth factor (VEGF) and placental growth factor (PlGF) play an important role in facilitating angiogenesis in placenta and transforming spiral arteries into low resistance capacitance vessels [71–73]. Failure of remodeling of spiral arteries by extravillous trophoblast is seen in placentae from pregnancies complicated by pre-eclampsia or FGR and when there is an imbalance of proangiogenic (PlGF) and antiangiogenic factors [soluble fms-like tyrosine kinase 1 (sFlt-1)] [72]. Indeed the 2021 International Society for the Study of Hypertension in Pregnancy (ISSHP) [74] now includes angiogenic factors as a criterion to define uteroplacental dysfunction (placental abruption, PlGF <5^th^ centile for gestational age or sFlt-1/PlGF ratio >38, FGR, abnormal umbilical artery Doppler waveform analysis or intrauterine fetal death).

### PlGF, sFlt-1, and sFlt-1/PlGF ratio

In a recent publication, Gaccioli et al. [75] showed that although the sFlt-1/PlGF ratio was increased in both pre-eclampsia and FGR in both placenta and maternal serum, in pre-eclampsia the sFlt-1/PlGF ratio was strongly associated with placental sFlt-1 concentrations (*r*=0.45; *P*<0.0001) but not placental PlGF concentrations (*r*=−0.17; *P*=0.16). In FGR pregnancies, however, the sFlt-1/PlGF ratio was strongly associated with placental PlGF concentrations (*r*=−0.35; *P*=0.02) but not placental sFlt-1 concentrations (*r*=0.04; *P*=0.81) suggesting that in pre-eclampsia the elevated sFlt-1/PlGF ratio is primarily driven by increased placental sFlt-1, whereas in FGR, it is mainly due to decreased placental PlGF.

In a prospective cohort study [76], low plasma PlGF (<5^th^ percentile for gestational age) identified FGR infants and significant placental dysfunction on histopathological examination with sensitivity of 98.2% (95% CI: 90.5–99.9) and PPV of 58.5% (95% CI: 47.9–68.6), respectively. Low maternal PlGF outperformed gestational age, fetal abdominal circumference, and umbilical artery Doppler resistance indices in predicting FGR secondary to placental dysfunction. In another study, high sFlt-1 expression was present in 28% of placental tissue from pregnancies complicated by SGA/FGR without pre-eclampsia and in this group, 90% had abnormal umbilical Doppler and lower mean BW [77].

The sFlt-1/PlGF ratio is inversely correlated with BW [78,79] and a high ratio is present in pregnancies complicated by FGR [80]. Furthermore, although the sFlt-1/PlGF ratio is elevated regardless of the gestation at which FGR is diagnosed, early-onset FGR is associated with higher ratios compared with late-onset FGR, suggesting a possible lesser degree of placental dysfunction in the latter group [78,81]. A high sFlt-1/PlGF ratio also predicts a shorter time to delivery interval, which in turn is even more strongly correlated with the magnitude of daily increase of the ratio [82]. A recent study by Mitlid-Mork et al. [83] showed that compared with controls, women with pregnancies complicated by placental syndromes (pre-eclampsia and/or FGR) median maternal concentrations of PlGF (104 vs. 165 pg/ml) were significantly lower while sFlt-1 (6927 vs. 4371 pg/ml) and the sFlt-1/PlGF ratio (73.1 vs. 28.4) were significantly higher. In another study that evaluated 120 cases of early-onset FGR, 75% had an sFlt-1/PlGF ratio ≥85 with an associated probability of delivery within 1 week of diagnosis of 36%. In contrast, a ratio of <85 was associated with a >70% probability of prolongation of pregnancy for >4 weeks [84]. A more recent study of early-onset FGR demonstrated a negative predictive value (NPV) (using an sFlt-1/PlGF cutoff threshold of 38) of 100% (95% CI: 0.92–1.00) for delivery within 2 weeks of diagnosis and a NPV of 50% for delivery within 1 week if the ratio was >85 [85]. Gaccioli et al. [86] reported that using an EFW of <10^th^ centile for gestation and sFlt-1/PlGF ratio of >5.78 at 28 weeks resulted in a PLR of 41.1 (95% CI: 23.0–73.6) and PPV of 21.3% (95% CI: 11.6–35.8) for preterm birth of an SGA infant. Using a higher threshold sFlt-1/PlGF ratio of >38 at 36 weeks resulted in a PLR of 17.5 (95% CI: 11.8–25.9) for subsequent birth of an SGA infant associated with either maternal pre-eclampsia or perinatal morbidity or mortality [86]. Other observational studies have also reported similar associations between a high sFlt-1/PlGF ratio with SGA/FGR and a shorter duration to delivery interval [87,88]. A recent systematic review and meta-analysis (33 studies; 9426 women) showed that while PlGF, sFlt-1, and the sFlt-1/PlGF ratio showed promise for the prediction of adverse maternal and perinatal outcomes including SGA/FGR and time to delivery, PlGF was equivalent to the sFlt-1/PlGF ratio for predictive utility [89].

In a prospective study of 3953 singleton pregnancies at 35–37 weeks of gestation, Valino et al. showed that prediction of SGA (detection rate: 62.8%) was best achieved by maternal serum PlGF, ultrasound EFW, and UtA-PI [90]. However, compared with maternal serum PlGF, sFlt-1 does not provide significant independent prediction of SGA [90,91]. Another study by Valino et al. that screened 8268 singleton pregnancies at an earlier gestation of 30–34 weeks demonstrated that prediction of SGA using EFW, PlGF, sFlt-1, UtA-PI, umbilical artery pulsatility index, and middle cerebral artery pulsatility index resulted in a detection rates of 88% and 51% for birth of a preterm and term SGA infant, respectively [92].

A recent systematic review and meta-analysis (eight studies; 5450 women) [93] evaluating the diagnostic capacity of the sFlt-1/PlGF ratio for FGR showed that a ratio of >33 was predictive for FGR, [Sensitivity 63% (95% CI: 54–71), specificity 84%, (95% CI: 83–85)] but had a low PLR of 3.55 (95% CI: 1.98–6.34). A higher ratio of ≥85 resulted in higher sensitivity 79% (95% CI: 66–89) but with similarly low PLR of 3.23 (95% CI: 0.94–11.11). Given the clear correlation of elevated sFlt-1 and sFlt-1/PlGF ratio with placental dysfunction and SGA/FGR infants both in early [94–96] and late gestation [86,97,98], there is increasing evidence supporting their use together with maternal characteristics and fetal biophysical ultrasound parameters in screening tests for SGA/FGR [22,24,99,100]. A high sFlt-1/PlGF ratio also appears to be predictive of adverse neonatal outcomes (admission to neonatal intensive care unit, severe respiratory disorders, and necrotizing enterocolitis) in SGA neonates [101,102]. There is some evidence however that fetal sex may also influence the sFlt-1/PlGF ratio. In a recent study, normotensive women with male fetuses had significantly higher sFlt-1 concentrations and sFlt-1/PlGF ratio compared with normotensive women with a female fetus. However, this difference was not observed in pregnant women with hypertensive disorders [103]. In another study, the sFlt-1/SPINT1 ratio was significantly raised in pregnancies with pre-eclampsia and/or SGA with median ratios (IQR) of 1.4 [0.44–2.54] and 0.82 [0.28–1.39] for BW <3^rd^ and 3^rd^–10^th^ centiles, respectively [70].

Another retrospective cohort study [104] reported that a low sFlt-1/PlGF ratio of <23 ruled out early-onset pre-eclampsia between 24- and 33^+6^-weeks’ gestation (NPV of 100%), while a ratio of >45 in combination with N-terminal-pro b-type natriuretic peptide (NT-proBNP) concentrations of >174 pg/ml increased the PPV from 49.5% to 86% (95% CI: 79.2–92.6). The median concentrations of NT-proBNP were significantly higher in women with pre-eclampsia (156.5 pg/ml, IQR: [78–343]) compared with those with isolated FGR (48 pg/ml, IQR: [24–59]) and normal pregnancy (47.5 pg/ml, IQR: [25–89]) [105].

In twin pregnancies, the sFlt-1/PlGF ratio measured in the second trimester is associated with increased odds for FGR (OR: 39.6, 95% CI: 6.31–248.17) [106]. However, as a standalone marker, PlGF does not appear to be sufficiently robust (sensitivity 27%) for the prediction of FGR for women with multiple pregnancy [107].

### Soluble endoglin

Another placenta-derived antiangiogenic factor associated with placental dysfunction is soluble endoglin (sEng). sEng is a soluble transforming growth factor-β (TGF-β) coreceptor, which has been shown to be elevated in sera of women with pre-eclampsia and FGR [108–110]. An early small observational study (44 women) reported positive correlation between sEng and sFlt-1 concentrations (Pearson 0.653; *P*<0.05) with significantly higher sEng concentrations in FGR pregnancies compared with controls. However, concentrations of sEng were lower in FGR compared with pre-eclampsia pregnancies [111]. More recent study showed that sEng is strongly correlated with sFlt-1/PlGF ratio with higher concentrations observed in FGR (OR: 2.28, 95% Cl: 1.55–3.4 and 2.38, 95% CI: 1.64–3.44 for sEng and sFlt-1/PlGF, respectively) [112]. Another study, however, did not find any significant association between maternal concentrations of sEng and time of delivery in pregnancies complicated by FGR [113]. The current evidence for the utility of sEng for the prediction of SGA/FGR is limited.

## Cell-free fetal DNA

Circulating cell-free fetal DNA (cffDNA) is used for aneuploidy screening, determination of fetal red cell antigen status, fetal sex, and screening for single-gene disorders [114–116]. cffDNA concentrations increase with gestational age and significantly higher levels are seen pregnancies complicated by placental dysfunction [117,121]. The data however from pregnancies complicated by FGR are conflicting, with some studies suggesting an increase [117–119] in cffDNA concentrations while others showing a decrease [120–122] compared with controls.

Lower median cffDNA fractions were observed only in women with early but not late FGR [121,122], suggesting that the lower fetal fraction could be the consequence of a smaller placental mass. However, other studies report that cffDNA concentrations are increased in pregnancies complicated by FGR with abnormal umbilical artery Doppler velocimetry raising the possibility that fetal DNA release is associated more with chronic fetal hypoxia than with fetal size [123]. Caramelli et al. [118] reported a more than twofold increase in cffDNA concentration in pregnancies complicated by FGR and abnormal uterine artery Doppler waveforms when compared with controls [117]. In another analysis, Smid et al. [119] showed that maternal plasma fetal DNA concentration in pregnancies complicated by FGR, median cffDNA concentrations were higher compared with controls (308.1 vs. 74.8 g.e./ml).

Poon et al. [124] measured plasma cffDNA from 1949 singleton pregnancies at 11–13 weeks of gestation and found that although concentrations were inversely related to maternal weight and UtA-PI, compared with controls, there was no difference with pregnancies complicated by SGA/FGR [124]. Other observational studies have also reported the lack of difference between cffDNA concentrations in FGR and control cohorts [125].

In a retrospective cohort study of 4317 singleton pregnancies [120], the fetal fraction was inversely correlated with MAP, UtA-PI, and positively associated with maternal PAPP-A and PlGF concentrations. A lower fetal fraction was associated with a higher risk of preterm FGR. Given the limited and inconsistent data regarding the relationship between maternal cffDNA concentrations and SGA/FGR, its utility as a reliable predictive marker remains unclear and further research is required [126–128].

## MicroRNAs

MicroRNAs (miRNAs) are small nonprotein-coding, single-stranded RNA molecules of up to 19–25 nucleotides. They influence post-transcriptional gene expression and help regulate cell development, differentiation, proliferation, and apoptosis [129,130]. Because they are relatively stable and resistant to degradation by temperature and pH changes circulating miRNAs have potential as biomarkers for the prediction of adverse placenta-related outcomes [131].

The placenta expresses many generic as well as placenta-specific miRNAs, which influence angiogenesis as well as trophoblast differentiation, proliferation, invasion, and migration [132]. Placentally derived miRNAs are exported from syncytiotrophoblast cells into the maternal circulation via exosomes [133]. Table 2 [134–140] details currently known circulating miRNAs associated with FGR.

**Table 2:** Circulating miRNAs in FGR

miRNA type	Expression in FGR/SGA	References
miR-210	Increased	[140]
miR-21	Increased	[140]
miR-424	Increased	[140]
miR-199a	Increased	[140]
miR-20b	Decreased	[137]
miR-942-5p	Decreased	[137]
miR-324-3p	Decreased	[137]
miR-127-3p	Decreased	[137]
miR-223-5p	Decreased	[137]
miR-17-5p	Decreased	[134]
miR-146a-5p	Decreased	[134]
miR-574-3p	Decreased	[134]
miR-221-3p	Decreased	[134]
miR-374a-5p	Increased	[136]
Let-7d-5p	Increased	[136]
miR-191-5p	Increased	[136]
miR-107	Decreased	[136]
miR-30e-5p	Decreased	[136]
miR-4454+7975	Decreased	[136]
miR-27b-3p	Increased	[138]
miR-16-5p	Increased	[138]
miR-103-3p	Increased before 32 weeks of gestation Decreased between 32 and 37 weeks of gestation	[138]
miR-107-3p	Increased before 32 weeks of gestation Decreased between 32 and 37 weeks of gestation	[138]
miR-346	Increased	[139]
miR-582-3p	Increased	[139]
miR-16-5p	Increased	[135]
miR-20a-5p	Increased	[135]
miR-146a-5p	Increased	[135]
miR-155-5p	Increased	[135]
miR-181a-5p	Increased	[135]
miR-195-5p	Increased	[135]
miR-145-5p	Increased	[135]
miR-342-3p	Increased	[135]
miR-574-3p	Increased	[135]
miR-1-3p	Increased	[135]
miR-20b-5p	Increased	[135]
miR-126-3p	Increased	[135]
miR-130b-3p	Increased	[135]
miR-499a-5p	Increased	[135]

**Table 3:** Essential miRNAs in pre-eclampsia with or without FGR

Pre-eclampsia with FGR	Pre-eclampsia without FGR
miR-210	miR-144
miR-17	miR-152
miR-16	miR-182
miR-21	miR-29a
miR-103	miR-29b
miR-181a	miR-24
miR-130b-3p	miR-26a
miR-155	miR-299
miR-181a	miR-342-3p
miR-20a	miR-215
miR-20b	miR-650
miR-126	miR-423-5p
miR-519a	miR-629-5p
miR-141	miR-18a
miR-194	miR-195
miR-520a-5p	miR-376c
miR-525	
miR-146a-5p	
miR-221-3p	
miR-574-3p	
miR-346	
miR-582-3p	
miR-126	

miR-210, a hypoxia-induced miRNA is expressed in different subtypes of placental trophoblasts and its deficiency is causally related to pre-eclampsia and placental adaptation to maternal hypoxia [141–143]. In pregnancies complicated by FGR, decreased expression of some placenta-specific miRNAs (miR-21, miR-16, miR-516b, miR-518b, miR-520h, miR-526b, miR-515-5p, miR-519d, and miR-1323) [144,145] have been reported. Table 3 lists the essential miRNAs in pre-eclampsia with or without FGR. miR-16 (OR: 4.13, 95% CI: 1.42–12.05) and miR-21 (OR: 2.43, 95% CI: 0.93–6.37), in particular, are strongly associated with birth of an SGA infant [144]. However, in another study, although four specific miRNAs (has – miR-518b, has – miR-1323, has – miR-520h, and has – miR-519d) were confirmed as FGR-associated, placenta-specific miRNAs, there was no difference in maternal plasma concentrations between FGR and uncomplicated pregnancies [145].

Whitehead et al. [140] found three- to sixfold increased concentrations of miR-210, miR-424, miR-21, miR-199a, and miR-20b in women with severe preterm FGR, which correlated with ultrasound Doppler velocimetry. On the other hand, higher circulating maternal serum concentrations of miR-20b-5p, miR-324-3p, miR-223-5p, and miR-127-3p in the second trimester were associated with lower odds of having an SGA infant [137]. Hromadnikova et al. [146] showed that in pregnancies complicated by FGR, significantly decreased concentrations of seven miRNAs were seen: miR-100-5p, miR-125b-5p, miR-199a-5p, miR-17-5p, miR-146a-5p, miR-221-3p, and miR-574-3p. Kim et al. [136] identified two unique miRNAs (hsa-miR374a-5p and hsa-let-7d-5p) that were expressed in significantly higher concentrations in plasma of women with SGA infants, indicating their potential for early prediction of SGA/FGR. Another recent study by Hromadnikova et al. [135] assessed the association of 29 cardiovascular disease-associated miRNAs in first trimester maternal blood samples and found that concentrations of six miRNAs were significantly increased in SGA/FGR pregnancies: miR-16-5p, miR-20a-5p, miR-146a-5p, miR-155-5p, miR-181a-5p, and miR-195-5p. A combination of four miRNAs (miR-1-3p, miR-20a-5p, miR-146a-5p, and miR-181a-5p) detected almost 76% of SGA infants, while a combination of seven miRNAs (miR-16-5p, miR-20a-5p, miR-145-5p, miR-146a-5p, miR-181a-5p, miR-342-3p, and miR-574-3p) detected approximately 43% of FGR infants [135].

Tagliaferri et al. [138] evaluated a group of hypoxia-regulated miRNAs and found elevated circulating concentrations of miR-16-5p, miR-103-3p, miR-107-3p, and miR-27b-3p in early FGR (<32 weeks of gestation), while reduced concentrations of miR-103-3p and miR-107-3p were noted in late FGR (measured between 32 and 37 weeks of gestation). Kim et al. [136] assessed 50 miRNAs profiles across gestation in SGA (defined as BW < 5^th^ percentile) pregnancies and found significantly increased maternal plasma concentrations of miR-374a-5p, let-7d-5p, and miR-191-5p and decreased concentrations of miR-107, miR-30e-5p and miR-4454+7975. Of these miRNAs, miR-374a-5p and let-7d-5p showed reasonable predictive value for SGA when evaluated individually (AUROC: 0.71, 95% CI: 0.56–0.86 and 0.74, 95% CI: 0.55–0.93), respectively, with improvement when both were combined (AUROC 0.772, 95% CI: 0.601–0.943) [136]. Although there are some specific miRNAs that are associated with placental dysfunction, which may have a role to play for either the prediction or diagnosis of FGR, their utility thus far, as reliable clinical biomarkers is uncertain.

## Conclusions

Early prenatal identification of infants at high risk of SGA/FGR or adverse perinatal outcomes such as stillbirth, neonatal morbidity, and mortality is important because it potentially allows decisions regarding intensity of antenatal surveillance, timing of birth, model of maternity care, parental counselling, and co-ordination of neonatal resources to be made. Thus, the attraction of a simple and acceptable screening test early in pregnancy is obvious. However, there are several circulating biomarkers that are clearly associated with adverse outcomes, none have yet, either alone or in combination, been shown to be sufficiently reliable to be used in clinical practice [147]. Some, such as PlGF [148] and sFlt-1 [149] show the most promise but require further validation to determine their screening performance. More importantly, however, it is important to determine if a policy of screening for disorders related to placental dysfunction results in improvements in clinical outcomes.

## Data Availability

All data are included within the main article.

## Open Access

Open access for the present article was enabled by the participation of University of Queensland in an all-inclusive *Read & Publish* agreement with Portland Press and the Biochemical Society under a transformative agreement with CAUL.

## Abbreviations

β-hCG: beta-human chorionic gonadotrophin
ADAM12: a disintegrin and metalloprotease 12
AFP: alpha-fetoprotein
AUROC: area under receiver-operating curve
BW: birthweight
cffDNA: cell-free fetal DNA
EFW: estimated fetal weight
FGF: fibroblast growth factor
FGR: fetal growth restriction
FPR: false-positive rate
IGF-I: insulin-like growth factor-I
IGFBP-1, -3, -4: IGF-binding protein-1, -3, -4
IQR: Interquartile range
LBW: low birth weight
MAP: maternal mean arterial pressure
MoM: multiples of median
N-CAM: neural cell adhesion molecule
NLR: negative likelihood ratio
NPV: negative predictive value
NT-proBNP: N-terminal-pro b-type natriuretic peptide
OR: odds ratio
PAPP-A: pregnancy-associated plasma protein-A
PGH: placental growth hormone
PIGF: placental growth factor
PLR: positive likelihood ratio
PP13: placental protein 13
PPV: positive predictive value
RR: relative risk
sEng: soluble endoglin
sFlt-1: soluble fms-like tyrosine kinase 1
SGA: small for gestational age
SPINT1: serine protease inhibitor Kunitz type 1
TGF-β: transforming growth factor-beta
UtA-PI: uterine artery pulsatility index
VEGF: vascular endothelial growth factor
